# MPVT+: a noise-robust training framework for automatic liver tumor segmentation with noisy labels

**DOI:** 10.3389/fmed.2025.1653865

**Published:** 2025-09-10

**Authors:** Xuan Cheng, Haoxiang Tian, Jiajun Zhou, Tianshu Xie, HaiGang Gong, Ming Liu, Yi Wei, Wei Lu

**Affiliations:** ^1^University of Electronic Science and Technology of China, Chengdu, China; ^2^Yangtze Delta Region Institute (Quzhou), University of Electronic Science and Technology of China, Quzhou, China; ^3^The Quzhou Affiliated Hospital of Wenzhou Medical University, Quzhou People's Hospital, Quzhou, China; ^4^Department of Radiology, West China Hospital, Sichuan University, Chengdu, China

**Keywords:** liver tumor segmentation, noisy label, semi-supervised learning, deep learning, annotation

## Abstract

**Introduction:**

Liver cancer is among the leading causes of cancer-related deaths worldwide. Accurate delineation of hepatic tumors is crucial for diagnosis, prognosis, and treatment planning, yet manual annotation is labor-intensive and subject to variability. Deep neural networks (DNNs) have shown promise in automating segmentation but require large amounts of high-quality labeled data, which is difficult to obtain. Incorporating noisy labels without proper handling can corrupt training and degrade performance.

**Methods:**

We introduce MPVT+, a noise-robust training framework that integrates a pixel-wise noise-adaptation module with a multi-stage perturbation and variable-teacher (MPVT) consistency strategy. The noise adaptor infers corruption probabilities and re-weights unreliable supervision, while MPVT assembles an ensemble of stochastic teacher models that apply progressively stringent perturbations. This combination enables the network to exploit both clean and noisy labels without overfitting.

**Results:**

Experiments conducted on 739 retrospectively collected liver-tumor CT datasets demonstrated that MPVT+ significantly outperformed baseline and traditional noise-handling approaches. Compared to a noise-free U-Net baseline (Dice Similarity Coefficient [DSC] 75.1%), MPVT+ improved segmentation accuracy to 80.3%. The framework consistently achieved superior results across multiple evaluation metrics, including DSC, JSC, SVD, and VOE.

**Discussion:**

The MPVT+ framework demonstrates that principled noise modeling, coupled with consistency training, effectively unlocks the potential of imperfect medical datasets. This strategy reduces the dependency on perfectly labeled datasets and moves fully automated liver tumor delineation closer to clinical applicability.

## 1 Introduction

Liver cancer is a prevalent malignant tumor and ranks as the third leading cause of cancer-related deaths globally in 2020 (1). An annual rise in liver cancer cases is noted, making it the only cancer among the top five deadliest (2). Diagnosing liver cancer typically involves various medical imaging techniques such as ultrasound, magnetic resonance imaging (MRI), and computer tomography (CT). Among these, CT is the most commonly used method because of its multiphasic contrast enhancement capabilities, providing more advanced imaging modalities (3). However, accurate diagnosis and subsequent treatment planning rely heavily on precise delineation of the tumor boundaries within the liver. The physical characteristics of a tumor, such as its size, shape, and location, are important biomarkers that play a key role in accurate diagnosis and treatment (4).

Deep learning has shown great potential in medical segmentation tasks (5, 6). However, the quality of labels used for training is particularly concerning (7, 8) as it directly impacts model performance of correctly discerning and segmenting tumors and consequently influences clinician' decisions of diagnoses and treatments of tumors. Accurate, consistent, and clear labels not only enhance the performance and generalization ability of the model but also ensure that the segmentation results possess clinical interpretability and application reliability. Primarily, erroneous label data can impact the performance of deep learning models, potentially leading to the learning of incorrect features or segmentation boundaries. Second, noisy labels can affect the model's generalization ability, resulting in poor performance on unseen data (9). In addition, overfitting to noisy labels can cause a decline in the model's performance on test data as it fails to accurately capture the true distribution of the data. Finally, the presence of low-quality labels increases the difficulty of training and requires corresponding strategies to mitigate their impact, to enhance the model's performance and generalization ability. Therefore, ensuring high-quality label data is crucial for the success of tumor segmentation tasks.

To tackle the challenges posed by noisy labels, various strategies have been explored in this study. Some studies utilize annotations or consistent labels from numerous domain experts to improve the quality of data (10, 11). However, this method is impractical due to its significant financial and logistical resource consumption, which is not easily accessible. Moreover, inter-observer variability further compounds this issue as different radiologists may interpret ambiguous features differently based on their experience and training (12, 13). Other studies aim to reduce the workload of experts in medical image annotation by using computer assistance (14). However, variability among different observers and within the same observer cannot be disregarded when using this method, since a single radiologist may provide different annotations at different times, influenced by varying conditions such as fatigue or changes in perceptual criteria (15). Some studies utilize data automatically extracted from medical image databases such as hospital picture archiving and communication systems to create their training datasets (16, 17). Historical data may not always be available for every study and tend to be noisy. Some studies use crowd-sourcing to collect labeled data from non-experts (18, 19). However, they often do not produce labels of sufficient quality for general studies and typically have a high noise rate (19). Some researchers proposed to utilize noisy labels to enhance performance of models. These methods focus on creating a robust architecture to model latent parameters, enhance generalization, adjust loss functions, and select appropriate samples to reduce the impact of noisy labels on the model (9). For instance, some approaches leverage robust optimization techniques that specifically account for noise in the labels (20), some other proposed noise-robust loss functions aiming to lessen the influence of incorrectly labeled data during the training process (21). In addition, there is a growing interest in the implementation of transfer learning, where models pre-trained on large, clean datasets are fine-tuned on smaller, noisier datasets, thereby capitalizing on the learned representations that are less susceptible to label noise (22). Techniques such as label smoothing (23) and uncertainty modeling (24, 25) can also help the model to not overfit to the noisy labels and instead focus on the underlying pattern in the data (26). Some methods completely ignore the noisy labels and train the networks using a semi-supervised approach. These evolving methodologies underscore the dynamic nature of deep learning in medical images, where ongoing research continually refines the balance between data quality and model reliability.

Probabilistic noise model is a fundamental concept in robust architecture. These methods are founded on the concept that noise can be represented by latent variables using neural networks (9, 27). Our method is inspired by both robust architecture and semi-supervised learning. To train deep neural network with noisy labels, it is crucial to identify noisy labels and eliminate the negative impact of them as much as possible. Based on the concept of noise adaptation layer (28), we introduced noise adaptor which alters convolutional neural networks by adding fully connected layers and softmax layers to predict hidden variables and simulate the generation of label noise. This module dynamically adjusts during training and refurbish the unreliable pixels in labels to produce unbiased supervisory signal. Furthermore, to prevent the model from overfitting due to potential changes in the data distribution, a semi-supervised framework called MPVT is implemented. This framework includes mean teachers with variable parameters updating inspired by dropout (29) to generate more robust pseudo-label guidance, which then allow us to introduce more perturbations into the training process to enhance the robustness and stability of the student model. This process effectively increases the model's resilience to overfitting by presenting it with a variety of training scenarios, ensuring that it learns to generalize from the core features of the data rather than its noisy aspects. This framework aims to help the model learn from reliable data and extract valuable information from noisy labels without being affected by the noise. Compared with previous methods, our method not only identifies and reduces the influence of noisy labels but also reinforces the robustness of the network by harnessing the inherent uncertainty within the data. This dual strategy effectively creates a noise-robust learning process, thereby improving the quality of the model's predictions and ensuring greater reliability in clinical applications.

## 2 Materials and methods

### 2.1 Datasets acquisition

This retrospective cross-sectional study received approval from the institutional review board at West China Hospital of Sichuan University. It primarily focused on patients diagnosed with hepatocellular carcinoma (HCC), intrahepatic cholangiocarcinoma (ICC), or liver metastasis (MET). Due to the retrospective nature of the study, patient consent was waived. The objective was to analyze imaging datasets derived from patients who underwent CT screening at our institution. To maintain scientific rigor, patients included in the study were required to meet specific inclusion criteria: 1. Patients had to be at least 18 years old to ensure that the research subjects exhibited adult physiological characteristics, thereby enhancing the relevance of our findings to the adult population. 2. Patients with a history of hepatectomy, transarterial chemoembolization, or radiofrequency ablation prior to the CT examination were excluded to eliminate potential confounding factors that could affect the experimental outcomes. 3. All included patients had confirmed diagnoses of HCC, ICC, or MET, either through pathology reports or follow-up imaging studies lasting at least 6 months, verified by at least two independent imaging methods. This stringent criterion aimed to ensure the accuracy and reliability of the tumor diagnoses.

**Figure 1:**
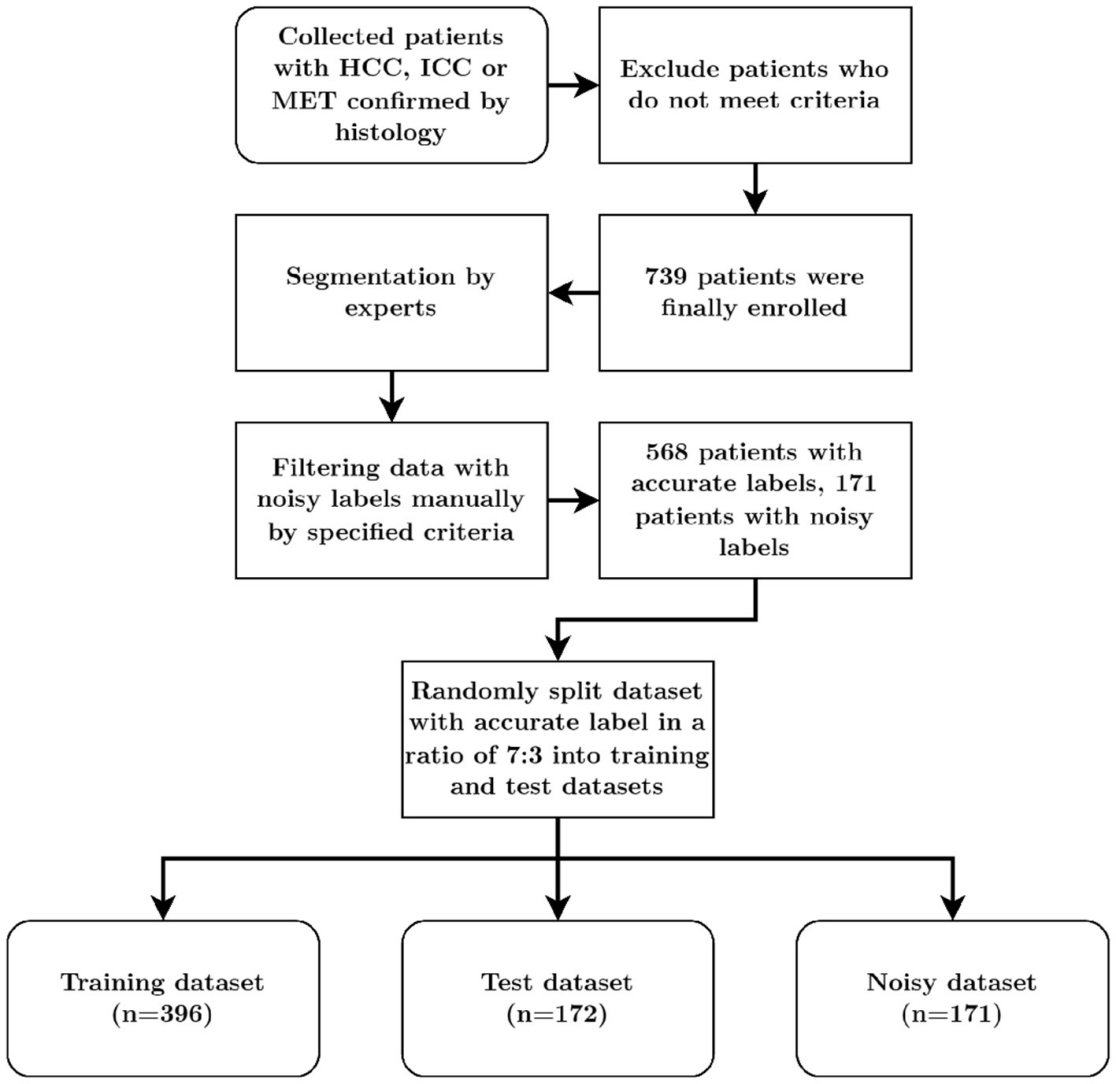
Dataset acquisition process.

Furthermore, to identify data with noisy labels, several specific criteria were established for CT images of patients: 1. Cases with labels that are visibly misaligned with the anatomical structures they are supposed to represent should be marked. 2. Cases where labels bleed into adjacent structures due to segmentation errors should be marked. 3. Cases with labels that are inconsistent across slices or with conflicting boundaries should be marked. 4. Cases with labels that contain random noise or are not representative of the true anatomy should be marked. 5. Cases where the labeled regions are incomplete or cutoff should be marked. In this study, a total of 739 patients were included, with further details provided in Figure 1.

### 2.2 Overall structure

To address the detrimental impact of noisy labels on network training, we introduced a module termed the noise adaptor. This module is designed to mitigate the disturbances caused by noise. Furthermore, we proposed a consistency regularization scheme, Multi-Stage Perturbations and Variable Teachers (MPVT), to effectively harness the latent information present in the CT images. The noise adaptor module, akin to the decoder of U-Net (30), generates probabilities of mislabeling. These probabilities enable the prediction of the likelihood of mislabeling or the introduction of noise to good labels. This information, when used in conjunction with the noisy labels, aids the supervised learning process. The noise adaptor seeks to extract a clean supervisory signal from the noisy labels by simulating noise, allowing the backbone network to learn the true data distribution more effectively, thereby enhancing the model's generalization ability and robustness.

Considering the significant impact of semi-supervised learning on model performance in the absence of accurate labels, we proposed a model that employs consistency regularization with multi-stage perturbations and variable teachers. This combined approach enhances the model's ability to cope with noisy labels and optimizes the use of input images by introducing various perturbations using different teacher models ensemble from different stages of training. We integrated the noise adaptor and MPVT scheme into the training process. Consequently, the overall training loss is:


(1) $$\begin{array}{l} \begin{array}{c} L=L_{sup}\left(D_{c}\right)+\beta_{ada}\left(L_{ada}\left(D_{n}\right)+\beta_{reg}\sigma \right)\\ +\beta_{semi}\left(L_{semi}\left(D_{c}\right)+L_{semi}\left(D_{n}\right)\right), \end{array} \end{array}$$

where $D_{c}$ and $D_{n}$ represent datasets with accurate labels and noisy labels, respectively; $L_{sup}$, $L_{ada}$, and $L_{semi}$ denote the supervision loss, adaptor loss, and semi-supervised loss, respectively; β_*ada*_ is the weight that determines the extent of the noise adaptor's supervision in training; σ represents the penalty term, and β_*reg*_ is the weight of the penalty term, which penalizes the noise adaptor to prevent it from finding shortcuts and not performing its supervisory role during training; β_*semi*_ is the weight of the semi-supervised learning component, and its magnitude is critical for the model's accuracy and generalization capability.

### 2.3 Noise adaptor module

To develop robust architecture that can adapt to label noise, it is essential to identify and model the types of label noise. Label corruption can be caused by the characteristics of images and labels themselves, which may increase inter- and intra-observer variation due to ambiguity that can prevent annotators from behaving consistently. In addition, random perturbation should be considered as the labeling process cannot be consistent and ideal.

Assuming that the corruption process is conditionally independent of label categories, label noise can be categorized as symmetrical and asymmetric noise. Symmetrical noise, or pure random noise, occurs when labels are corrupted by label transition probabilities *p*_*ij*_, where *i* is the true label and *j* is the corrupted label. This type of noise is termed symmetrical because the probabilities of flipping a true label into any other labels are equal, which can be expressed as follows:


(2) $$\begin{array}{l} \left(\exists \rho \in \left[0,1\right]\right)\wedge \left(\forall_{i=j}p_{ij}=1-\rho \right)\wedge \left(\forall_{i\neq j}p_{ij}=\frac{\rho }{1-c}\right), \end{array}$$

where ρ is the noise rate, and *c* is the number of classes. This equation reflects the equal likelihood of a label being incorrectly assigned to any other class in the presence of symmetrical noise;


(3) $$\begin{array}{l} \left(\exists \rho \in \left[0,1\right]\right)\wedge \left(\forall_{i=j}p_{ij}=1-\rho \right)\wedge \left(\forall_{i\neq j}\overset{c}{\underset{j=0}{\sum }}p_{ij}=\rho \right), \end{array}$$

where the probabilities of a true label being mislabeled as another one differ from one another. This implies that the true label is most likely to be mislabeled as a particular label. To implement this concept, we introduce a learnable layer at the end of the segmentation network output, inspired by Goldberger and Ben-Reuven (28).

In more realistic scenarios, label corruption is assumed to be related to both labels and features. This type of label noise is referred to as instance-dependent label noise (27, 28). The probability with noise can be formulated as:


(4) $$\begin{array}{l} p_{ij}\left(x\right)=p\left(ỹ=j|y=i,x\right), \end{array}$$

where *x* represents the input to the networks. This implies that, in addition to the aforementioned noises, the features of the input data also have an impact on the annotations. For instance, in some images, the form of the portal vein may resemble a lesion, making it more likely to be mistaken as a tumor.

However, in the context of segmentation, there are differences compared to classification. Segmentation involves pixel-wise classification, where positional factors must be taken into account. Therefore, we modified the noise adaptation layer based on the concept that different pixels should have different probabilities. For instance, the edges around CT images are likely to have low signal intensity as patients are typically positioned in the middle of the images. Conversely, distinct areas with signal intensity differing from their surroundings are more likely to be lesions. This concept is referred to as positional label noise, and it can be expressed as:


(5) $$\begin{array}{l} p_{ij}\left(\omega \right)=p\left(ỹ=j|y=i,\omega \right), \end{array}$$

where ω represents a particular pixel in the images.

**Figure 2:**
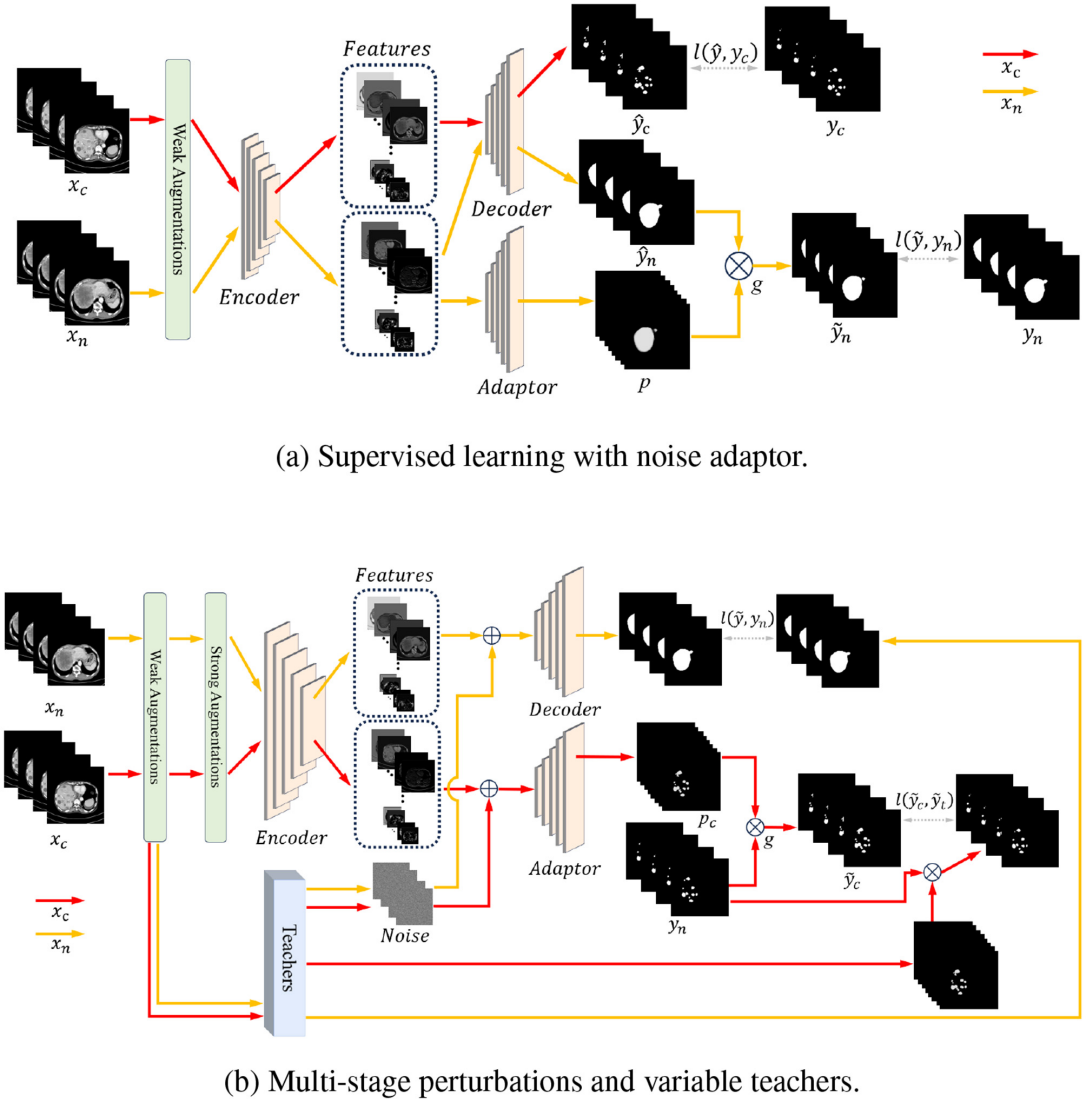
Overall structure diagram. **(a)** In the training process for supervised learning with the proposed noise adaptor, the data flow is represented by two distinct lines: The red line indicates the data flow of data with accurate labels, while the orange line represents the data flow of data with noisy labels. After the encoder processes the data, the features of data with noisy labels are fed into both the decoder and the adaptor. The adaptor then generates predicted noisy labels, which provide a supervisory signal for network training. **(b)** Consistency regularization is conducted with data that has accurate labels, while data with noisy labels are regarded as unlabeled data. This approach is used to enhance both the backbone networks and adaptor networks. By employing multi-stage perturbations and variable teachers, the consistency regularization process helps to improve the robustness and generalization capability of the model.

As depicted in Figure 2, we have implemented a noise adaptor based on the U-Net architecture (30). The adaptor is a decoder similar to the original U-Net decoder, receiving the extracted features from the encoder, which is part of the U-Net architecture. Then, the adaptor uses a single, direct convolutional layer (3 × 3 conv with 2 output channels) followed by a sigmoid layer to produce 2-channel probabilities. The 2-channel output represents the two probability endpoints that are used for linear interpolation with the main network's predictions. Through these two decoders, we obtain the predicted probabilities from the U-Net and the predicted corruption probabilities from the adaptor. The corruption probabilities consist of two parts: the probabilities of mislabeling background as foreground and the probabilities of correctly labeling foreground. We can then obtain the predicted probabilities with label noise according to the law of total probability, which can be formally expressed as:


(6) $$\begin{array}{l} {ŷ}_{ada}=ŷ\cdot p_{ada}\left(j=1|i=1\right)+\left(1-ŷ\right)\cdot p_{ada}\left(j=1|i=0\right), \end{array}$$

where ŷ_*ada*_ represents the prediction after adaptation, ŷ is the prediction of the U-Net, *p*_*ada*_(*j* = 1|*i* = 0) is the probabilities of mislabeling background as foreground, and *p*_*ada*_(*j* = 1|*i* = 1) is the probabilities of correctly labeling foreground. This process can be viewed as the corruption process from accurate labels to noisy labels. By employing this approach, we can train the model with data containing noisy labels in a normal supervised fashion.

To implement the aforementioned concept, we define the training loss for the network with the noise adaptor as:


(7) $$\begin{array}{l} L=L_{sup}+\beta_{ada}\left(L_{ada}+\beta_{reg}\sigma \right). \end{array}$$

This loss consists of two terms: the supervised loss and the adaptor loss. The supervised loss is denoted as:


(8) $$\begin{array}{l} L_{sup}=\frac{1}{\left|D_{n}\|\Omega \right|}\underset{\left(\text{x},\text{y}\right)\in D_{n}}{\sum }\underset{\omega \in \Omega }{\sum }l\left(f_{\theta }\left(T_{weak}\left(\text{x}\right)\right),T_{weak}\left(\text{y}\right)\right), \end{array}$$

where $D_{n}$ is the dataset with noisy labels, *l* is the loss function for supervised learning (we select cross-entropy loss and Dice loss), $T_{weak}$ represents weak augmentation (both images and labels are augmented with the same settings), and *f*_θ_ is the U-Net parameterized with θ. Ω denotes the set of pixels in each training sample over which the pixel-wise loss is computed. Since segmentation is formulated as pixel-wise classification, the summation over Ω ensures that the loss function accounts for every pixel in each training image. The adaptor loss is denoted as:


(9) $$\begin{array}{l} {ℒ}_{ada}=\frac{1}{\left|D_{n}||\Omega \right|}\sum_{(x,y)\in D_{n}}\sum_{\omega \in \Omega }l(g(f_{\theta }(T_{weak}(x)),\\ \;f_{\theta_{ada}}(T_{weak}(x))),T_{weak}(y)), \end{array}$$

where *f*_θ_*ada*__ is the adaptor and *g* is the transform function. According to the previous discussion, we define the transform function as:


(10) $$\begin{array}{l} \begin{array}{c} g\left(ŷ,p_{ada}\right)={ŷ}_{ada}=ŷ\cdot p_{ada}\left(j=1|i=1\right)\\ +\left(1-ŷ\right)\cdot p_{ada}\left(j=1|i=0\right). \end{array} \end{array}$$

In addition, a regularization term σ is added to prevent the adaptor from taking shortcuts and preventing the U-Net from learning anything. σ can be defined as:


(11) $$\begin{array}{l} \begin{array}{c} \sigma =\frac{1}{\left|D_{n}\|\Omega \right|}\underset{\left(\text{x},\text{y}\right)\in D_{n}}{\sum }\underset{\omega \in \Omega }{\sum }\|f_{\theta }\left(T_{weak}\left(\text{x}\right)\right)\\ -g\left(f_{\theta }\left(T_{weak}\left(\text{x}\right)\right)\right){\|}_{2}^{2}. \end{array} \end{array}$$

### 2.4 Multi-stage perturbations and variable teachers

Semi-supervised learning techniques are valuable for extracting features from unlabeled data, aiding neural networks in learning from it to enhance their overall generalization. When training a model with noisy labels, it can eventually become biased and suffer from poor generalization. By discarding noisy labels, the training process transitions to semi-supervised learning and fully utilizes the data with corrupted labels. Common techniques include consistency regularization via pseudo-labeling and perturbations. To implement this concept, we propose the MPVT framework–a self-training and consistency regularization framework with multi-stage perturbations and variable teachers–with reference to (31) and (32).

In the training process, the loss is defined as:


(12) $$\begin{array}{l} L=L_{sup}+\beta_{semi}L_{semi}, \end{array}$$

where $L_{sup}$ is the loss of supervised learning, and $L_{semi}$ is the loss of semi-supervised learning, which is calculated as:


(13) $$\begin{array}{l} {ℒ}_{ada}=\frac{1}{\left|D_{n}||\Omega \right|}\sum_{(x,y)\in D_{n}}\sum_{\omega \in \Omega }l(g(f_{\theta }(T_{weak}(x)),\\ \;f_{\theta_{ada}}(T_{weak}(x))),T_{weak}(y)), \end{array}$$

where $T_{strong}$ represents the strong augmentations, *f*_θ_*s*__ denotes the student model parameterized by θ_*s*_, $F_{\theta_{t}}$ is the ensemble of teacher networks, and ρ_τ_ is the function used to obtain pseudo-labels from predicted probabilities with thresholds τ_1_ and τ_2_. To address the sample selection bias that can affect the training process, a tailored pseudo-label generation method is proposed, formulated as:


(14) $$\begin{array}{l} y_{pseudo}\left(c\right)=1\left(ŷ\left(c\right)\geq \tau_{1}\vee \left(ŷ\left(c\right)\geq \tau_{2}\wedge y\left(c\right)=1\right)\right). \end{array}$$

In summary, we constructed a U-Net with a noise adaptor and trained it using the described training processes and losses. The main hyperparameters were as follows: we used Rectified Adam (RAdam) as the optimizer with an initial learning rate of 0.001 and a weight decay of 0.0001. Gradients were accumulated every 2 batches, with a batch size of 24 per worker. The learning rate was dynamically scaled down by a factor of 10 for epochs based on the current loss trend. We implemented the proposed model using PyTorch 1.2.0. The experiments were conducted on an Ubuntu 20.04.6 LTS server with two NVIDIA GeForce RTX 3090 cards.

### 2.5 Statistic indices

Here, we selected seven statistical indices to assess the performance of our method, including the Dice Similarity Coefficient (DSC), Jaccard Similarity Coefficient (JSC), Symmetric Volume Difference (SVD) and Volumetric Overlap Error (VOE)(33). These indices collectively provide a comprehensive evaluation of the method's accuracy, robustness, and efficiency in segmenting and quantifying the regions of interest.

DSC measures the overlap between two sets by calculating twice the intersection divided by the sum of the sizes of the two sets. JSC, also known as the Intersection over Union, is the ratio of the intersection of the sets to their union. SVD evaluates the symmetry of volume differences between two sets, providing insights into segmentation errors. VOE is the complement of the DSC and measures the volume not overlapping between two sets, often used to quantify segmentation accuracy.

To assess whether the proposed method achieved statistically significant improvements, we performed pairwise comparisons between the proposed method and other methods using a paired t-test on DSC, JSC, SVD, and VOE scores. The corresponding p-values were reported.

## 3 Data visualization and results

### 3.1 Negative impact of noisy label

**Figure 3:**
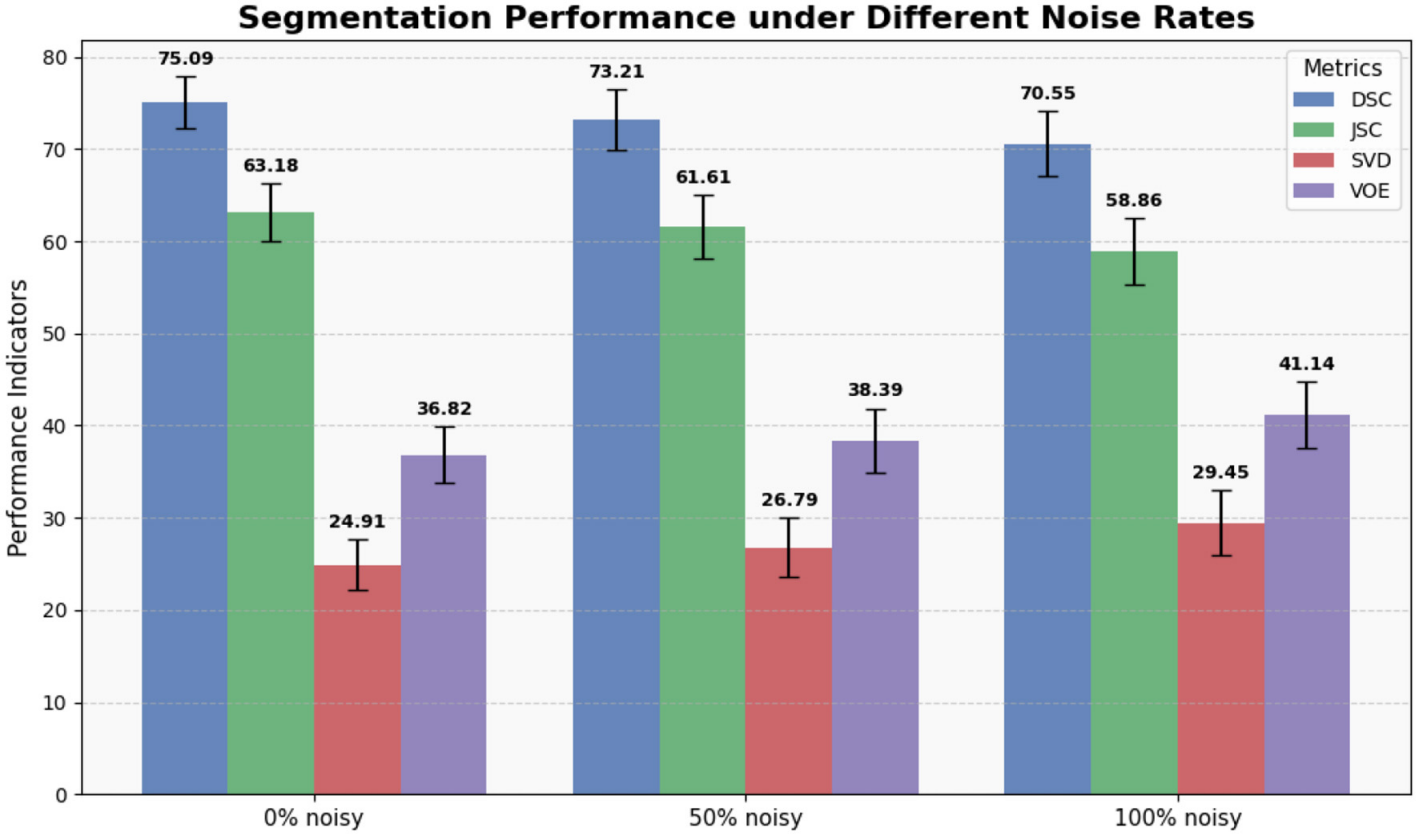
Display of results of segmentation performance under different noise rates.

Because introducing additional data with noisy labels may enhance the model's generalization ability, while noise also has the potential to impair generalization. Therefore, quantitatively analyzing the impact of noisy labels on the model becomes a pivotal aspect of our proposed noisy label handling framework. Hence, we conducted experiments with different noise rates (proportion of data with noisy labels in the training set) for evaluation. As shown in Table 1 and Figure 3, when the training data had a 0% noise rate, the U-Net with a ResNet34 (34) backbone achieved the best performance. The performance degraded with increasing noise rates, with the 50% and 100% noise rates resulting in the second and third-best performances, respectively. These results highlight that data with noisy labels can significantly impair model performance, regardless of the quantity utilized. Therefore, it is crucial to develop robust techniques for handling noisy labels in training datasets to ensure the reliability and effectiveness of machine learning models.

**Table 1:** Comparison of segmentation performance of different noise rates.

**Method**	**DSC (95% CI)**	***p*-value**	**JSC (95% CI)**	***p*-value**	**SVD (95% CI)**	***p*-value**	**VOE (95% CI)**	***p*-value**
ResNet34 (0% noisy)	**75.09** (72.30, 77.88)	-	**63.18** (60.07, 66.28)	-	**24.91** (22.12, 27.70)	-	**36.82** (33.72, 39.93)	-
ResNet34 (50% noisy)	73.21 (69.97, 76.46)	0.068	61.61 (58.18, 65.04)	0.093	26.79 (23.54, 30.03)	0.068	38.39 (34.96, 41.82)	0.092
ResNet34 (100% noisy)	70.55 (67.01, 74.09)	0.000	58.86 (55.24, 62.48)	0.000	29.45 (25.91, 32.99)	0.000	41.14 (37.52, 44.76)	0.000

The best results are bolded.

### 3.2 Contrastive analysis

**Figure 4:**
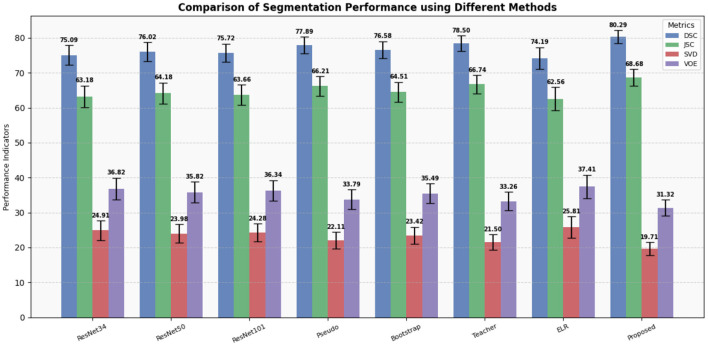
Display of results of segmentation performance using different methods.

To validate the superior performance of our proposed method, we conducted a comparative analysis against several traditional methods designed for handling data with noisy labels (31, 34–37). The summarized results of these comparisons are presented in Table 2. We also plot column charts to provide a more intuitive visual comparison of performance indicators, as demonstrated in Figure 4.

**Table 2:** Comparison of segmentation performance of different methods for liver tumors.

**Method**	**DSC (95% CI)**	***p*-value**	**JSC (95% CI)**	***p*-value**	**SVD (95% CI)**	***p*-value**	**VOE (95% CI)**	***p*-value**
ResNet34 (34)	75.09 (72.30, 77.88)	0.000	63.18 (60.07, 66.28)	0.000	24.91 (22.12, 27.70)	0.000	36.82 (33.72, 39.93)	0.000
ResNet50 (34)	76.02 (73.32, 78.71)	0.000	64.18 (61.18, 67.18)	0.000	23.98 (21.29, 26.68)	0.000	35.82 (32.82, 38.82)	0.000
ResNet101 (34)	75.72 (73.11, 78.34)	0.000	63.66 (60.72, 66.60)	0.000	24.28 (21.66, 26.89)	0.000	36.34 (33.40, 39.28)	0.000
Pseudo (35)	77.89 (75.50, 80.28)	0.011	66.21 (63.41, 69.01)	0.019	22.11 (19.72, 24.50)	0.010	33.79 (30.99, 36.59)	0.020
Bootstrap (36)	76.58 (74.12, 79.03)	0.000	64.51 (61.69, 67.33)	0.000	23.42 (20.97, 25.88)	0.000	35.49 (32.67, 38.31)	0.000
Teacher (31)	78.50 (76.28, 80.72)	0.041	66.74 (64.10, 69.38)	0.023	21.50 (19.28, 23.72)	0.045	33.26 (30.62, 35.90)	0.022
ELR (37)	74.19 (71.10, 77.28)	0.000	62.56 (59.24, 65.88)	0.000	25.81 (22.72, 28.90)	0.000	37.44 (34.12, 40.76)	0.000
Proposed	**80.29** (78.42, 82.17)	-	**68.68** (66.35, 71.00)	-	**19.71** (17.83, 21.58)	-	**31.32** (29.00, 33.65)	-

The best results are bolded.

Our experimental results clearly demonstrate that our method outperforms all other methods across various metrics, including DSC, JSC, SVD, and VOE. Particularly noteworthy are the improvements in DSC and JSC, which are key indicators of segmentation performance, where our method exhibits enhancements of 1.79% and 1.93%, respectively, compared to the best-performing alternative methods. Moreover, to assess the impact of data containing noisy labels on network performance, we conducted experiments using varying amounts of data with noisy labels.

Furthermore, to investigate whether the observed improvements are solely due to the increased model parameters, we compared the performance of ResNet34 with ResNet50 and ResNet101 (34). The results suggest that the modest increase in parameters marginally enhances the model's performance.

Therefore, our proposed method effectively leverages information from data with noisy labels, addressing the issue of deep learning models memorizing noisy information during training (38, 39), which can harm model performance. These findings suggest that our method can be a valuable tool in supporting the diagnosis of clinical liver tumors by providing auxiliary guidance.

**Figure 5:**
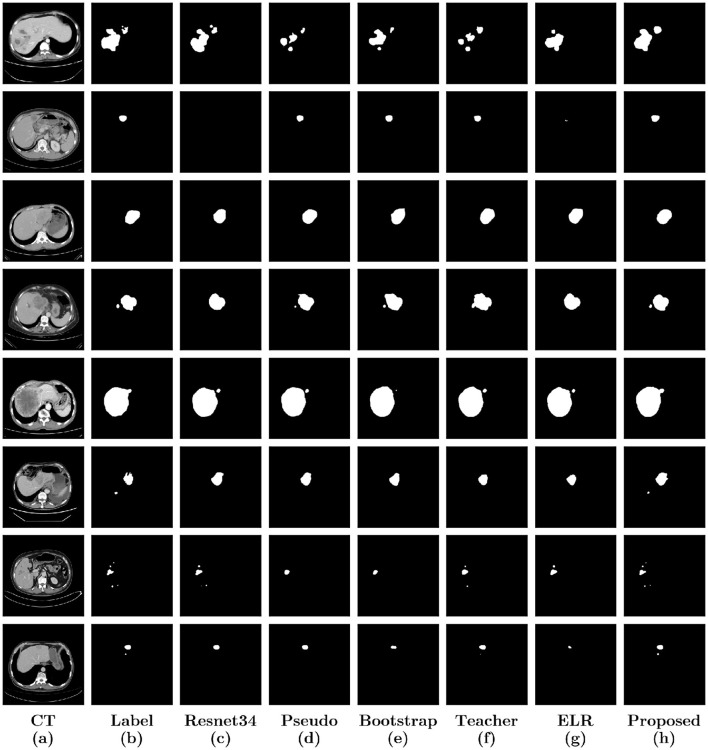
Display of results of liver tumor segmentation by different methods. Eight samples were selected to display the results of the six methods. **(a)** Original CT images; **(b)** Labels; **(c)** Results of U-Net with a backbone structure Resnet34; **(d)** Results of training with pseudo label; **(e)** Results of hard bootstrapping; **(f)** Results of training with mean teacher; **(g)** Results of training with ELR loss; **(h)** Results of our method.

For a more intuitive qualitative assessment, Figure 5 illustrates eight liver tumor segmentation cases, each column representing a different stage of the segmentation process. The first column shows the original CT image, followed by manual annotations by a doctor in the second column. The third column presents the segmentation results using U-Net with the ResNet34 backbone. Subsequent columns demonstrate the impact of different techniques on the segmentation results, including pseudo-labeling, hard bootstrapping, mean teacher integration, ELR method for preventing noisy label memorization, and finally, our proposed method. A detailed examination of these columns reveals the effectiveness of our proposed method in addressing issues such as false positives, under-segmentation, and coarse boundaries across various scenarios. Compared to alternative methods, our approach leads to notable improvements in liver segmentation accuracy.

### 3.3 Ablation analysis

To validate the effectiveness of our proposed method, we conducted an ablation study, the results of which are presented in Table 3. Our baseline model, depicted in the first row of Table 3, is the U-Net with backbone of ResNet34 trained using cross-entropy loss and dice loss. The ablation study focuses on evaluating the contributions of two key components: the noise adaptor (NA) and the Multi-Stage Perturbations and Variable Teachers framework (MPVT).

**Table 3:** Results of ablation analysis.

**Method**	**DSC (95% CI)**	**JSC (95% CI)**	**SVD (95% CI)**	**VOE (95% CI)**
ResNet34	75.09 (72.30, 77.88)	63.18 (60.07, 66.28)	24.91 (22.12, 27.70)	36.82 (33.72, 39.93)
Noise Adaptor	78.08 (75.92, 80.24)	66.08 (63.48, 68.68)	21.92 (19.76, 24.08)	33.92 (31.32, 36.52)
MPVT	79.27 (77.41, 81.13)	67.25 (64.92, 69.57)	20.73 (18.87, 22.59)	32.75 (30.43, 35.08)
w/o Input Perturbations	78.55 (76.28, 80.83)	66.85 (64.22, 69.49)	21.45 (19.17, 23.72)	33.15 (30.51, 35.78)
w/o Feature Perturbations	78.42 (76.30, 80.54)	66.44 (63.92, 68.97)	21.58 (19.46, 23.70)	33.56 (31.03, 36.08)
w/ One Teacher Model	77.92 (75.84, 80.00)	65.74 (63.20, 68.28)	22.08 (20.00, 24.16)	34.26 (31.72, 36.80)
Proposed Method	**80.29** (78.42, 82.16)	**68.68** (66.35, 71.00)	**19.71** (17.83, 21.58)	**31.32** (29.00, 33.65)

The best results are bolded.

The results of the ablation study indicate that each component contributes in a complementary manner to the overall performance. Specifically, the noise adaptor enhances accuracy by approximately 2.98% and 2.89% in terms of DSC and JSC, respectively, compared to the baseline model, indicating that the noise adaptor effectively learns label noise information, thereby assisting the backbone network in learning without the perturbation caused by noise, which helps prevent the network from memorizing noisy data.

Furthermore, the implementation of the MPVT scheme resulted in improvements of approximately 4.18% and 4.07% in DSC and JSC, respectively, compared to the baseline model. Particularly noteworthy is the significant improvement in sensitivity, suggesting that the consistency regularization enhances the robustness of the backbone network and leverages latent information present in the CT images. In addition, to validate the improvement of model performance by multi-stage perturbations and variable teachers, we conducted ablation experiments on each perturbation and teacher model variability, respectively. The experimental results indicate that performance declines when any stage of perturbation is missing during training, compared to the complete MPVT. This demonstrates that the enhancement of model robustness from each stage of perturbation is indispensable. Specifically, when the training process lacks the variability of the teacher model, the model's performance deteriorates the most. This underscores the necessity of using a variable teacher to enhance the model's adaptability to strict multi-stage perturbations for training. These results indicate that training the network with the MPVT scheme enhances the network's robustness.

**Figure 6:**
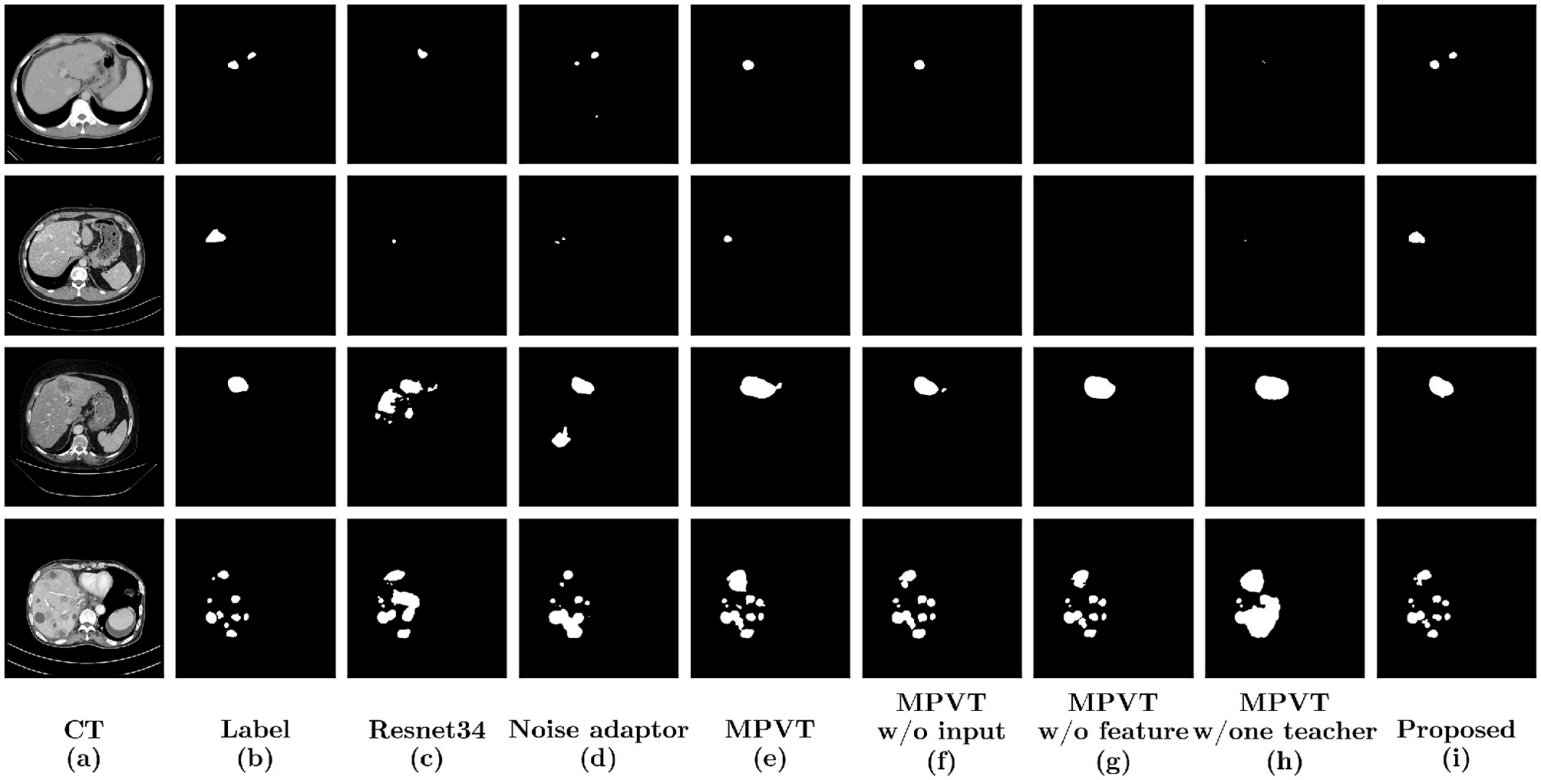
Display of results of ablation analysis. Four samples were selected to display the results of the six methods. **(a)** Original CT images; **(b)** Labels; **(c)** Results of U-Net with a backbone structure Resnet34; **(d)** Results of training with noise adaptor; **(e)** Results of MPVT; **(f)** Results of MPVT without input perturbations; **(g)** Results of MPVT without feature perturbations; **(h)** Results of MPVT with only one teacher model; **(i)** Results of our method.

In a comparative analysis, we have selected four cases depicted in Figure 6 to contrast seven distinct experiments. The results obtained from the baseline model exhibit a basic detection of lesion outlines and areas, with a tendency to mislabel regions resembling tumors and an inability to accurately identify all lesions. This limitation is attributed to the complex nature of liver CT images and the constraints of a limited training dataset.

In contrast, both the noise adaptor and MPVT approaches leverage additional data containing noisy labels, resulting in improved prediction quality. The noise adaptor tends toward conservative predictions, potentially overlooking some lesions, as seen in column 4 of Figure 6. On the other hand, MPVT leans toward bolder predictions, leading to over-expansion of the predicted area and mislabeling of other regions, as demonstrated in column 5 of Figure 6. Moreover, when multi-stage perturbations or variable teachers are removed, the model's ability to segment details and recognize interference areas decreases. This reflects the impact of lacking perturbations in the training process on the model's generalization ability and robustness.

Our proposed method combines the advantages of both approaches, adopting a compromise strategy that yields the most favorable results among the four methods. This suggests that our approach, utilizing training with noise adaptor and MPVT schemes, enables these two distinct strategies to mitigate each other's weaknesses and guide training outcomes toward a direction without bias.

## 4 Discussion

Automated liver-tumor segmentation remains hamstrung by the scarcity of perfectly annotated images and by the pernicious effect of label noise on deep neural networks. Here, we show that a multi-stage perturbation and variable-teacher (MPVT) consistency framework as well as a pixel-wise noise-adaptation module, termed MPVT+, enables convolutional networks to learn effectively from imperfect datasets, delivering state-of-the-art delineation accuracy. Across 739 retrospectively collected liver-tumor CT volumes, MPVT+ achieved the Dice similarity coefficient (DSC) from 75.1 % for a noise-free U-Net baseline to 80.3 %.

Various methods have been investigated to reduce the negative impact of noisy labels on medical image segmentation. For instance, obtaining annotations from multiple experts aims to establish a reliable ground truth but is limited by costs and remaining variability among annotators (10–13). Semi-automated tools can ease the annotation effort, although consistency issues among annotators still exist (14, 15). Utilizing historical hospital data provides scale but often suffers from inaccurate annotations (16, 17). Crowdsourcing involves more participants but typically introduces significant label noise (18, 19). To address noise during training, techniques such as designing robust models that account for latent noise parameters (9, 20), using custom loss functions to reduce the impact of incorrect labels (21), and applying transfer learning approaches that leverage knowledge from clean datasets (22) have been proposed. Additional regularization methods–such as label smoothing (23), help prevent the model from memorizing incorrect annotations (26). Building on these approaches, our MPVT+ framework integrates multiple components: a noise-adaptation module that explicitly models pixel-level corruption, multi-stage perturbations, and variable teachers that provide the network with self-supervised, noise-agnostic guidance. This combination advances liver-tumor segmentation beyond the performance of existing methods.

In addition, other recent studies have also emphasized the importance of robustness under imperfect or noisy annotations. Fang et al. (40) proposed a reliable mutual distillation framework, where two segmentation models collaborate to mitigate the impact of coarse or noisy labels through consistency constraints and sample selection strategies. Similarly, Qiu et al. (41) developed a hierarchical multimodal fusion framework that incorporates noisy label learning with attention mechanisms to enhance cancer classification performance from pathology and genomic data. Both works highlight complementary perspectives on leveraging imperfect supervision and noisy annotations, which aligns with the design philosophy of MPVT+ that explicitly models label corruption while enforcing consistency. These directions together underscore a growing consensus that noise-robust strategies are crucial for advancing reliable medical image analysis in real-world clinical scenarios.

In the broader context of semi-supervised medical image segmentation, recent work has emphasized the integration of consistency learning with uncertainty modeling. Zhang et al. (25) proposed an uncertainty-guided mutual consistency framework that leverages intra-task and cross-task regularization while filtering unreliable predictions through uncertainty estimation, thereby enhancing the exploitation of unlabeled data. More recently, (24) introduced an uncertainty-aware consistency learning strategy that incorporates multi-level perturbations and perturbation-based uncertainty estimation to suppress noisy predictions and improve generalization across diverse segmentation tasks. Compared with these approaches, the present MPVT+ framework addresses robustness from a complementary perspective: Instead of relying solely on uncertainty-guided filtering, MPVT+ explicitly models label corruption through a noise adaptor while simultaneously enforcing multi-stage perturbations and variable-teacher consistency. This dual strategy allows the model to effectively utilize both reliable and noisy supervision, mitigating the adverse effects of corrupted labels. Taken together, these studies suggest that uncertainty-guided consistency learning and explicit noise-robust adaptation are not mutually exclusive but potentially synergistic directions. Future research may benefit from combining these paradigms to further advance the reliability and clinical applicability of automated tumor segmentation systems.

The devised framework presents a suite of significant enhancements to traditional deep learning models. Primarily, it utilizes a noise adaptor module that refurbishing noisy pixels. This reduces the detrimental impact of label noise on the training process without compromising the quantity of training samples, ensuring that the model can be trained on a vast and diverse dataset, crucial for the development of a robust liver tumor segmentation tool. In addition, the proposed module might be used for understanding mechanisms of noise generation and how to effectively eliminate the negative impact of noisy labels. Second, the introduction of variable teachers equips the model with the capability to generalize better by preventing it from focusing on any special subset of datasets and perturbations during training, thereby reducing overfitting to corner cases and enable the model to be trained with more strict perturbations. Third, the semi-supervised learning framework, along with strict perturbations, trains the network to be robust against data variability and capable of extracting more general and robust features. This addresses the weakness of the noise adaptor being susceptible to perturbations that could degrade its performance. Furthermore, by synthesizing the strengths of robust architecture with the flexibility of semi-supervised learning, our method demonstrates superior utilization of noisy-labeled data, significantly elevating the model's performance. Finally, the adaptability of the framework ensures its suitability across various extents of noise intensity and types, augmenting its potential application in diverse medical imaging contexts.

However, there are some limitations of our study. We did not consider discrepancies between annotators during the training process, as obtaining annotations from multiple radiologists and pathologists is time-consuming. Future research will focus on incorporating factors such as the grading provided by each doctor to address label noise dependent on the annotator. In addition, while our implementation of the noise adaptor is effective, a more sophisticated architecture may be more adept at capturing intricate label noise patterns. Moreover, the use of multiple networks in the MPVT approach results in higher computational costs. Future research could explore training the network using a meta-learning framework to mitigate this issue. Finally, our study focused on scenarios where data with accurate labels could be differentiated from data with noisy labels, which can be a time-consuming process. Future research should investigate integrating mechanisms such as confidence curriculum learning to accelerate this data filtering process.

## 5 Conclusion

This study presents a novel deep learning framework that integrates a robust architecture with consistency regularization techniques to mitigate the adverse impact of noisy labels on DNNs training. By incorporating clean and noisy-labeled data, our approach enhances the model's robustness to noise and generalization capability, outperforming traditional methods and achieving better results. Our method shows promise for training segmentation CNNs tailored for detecting lesions in datasets affected by noisy labels. It effectively reduces the need for constructing a perfectly labeled dataset for DNNs training, serving as a valuable tool for aiding in clinical decision-making.

## Data Availability

The datasets presented in this article are not readily available because data from this study may contain potentially or sensitive patient information. Data are anonymized, nevertheless, due to relatively few severe cases, patients could be identified. Requests to access the datasets should be directed to Wei Yi, drweiyi057@163.com.
